# Generative AI–Enabled Therapy Support Tool for Improved Clinical Outcomes and Patient Engagement in Group Therapy: Real-World Observational Study

**DOI:** 10.2196/60435

**Published:** 2025-03-10

**Authors:** Johanna Habicht, Larisa-Maria Dina, Jessica McFadyen, Mona Stylianou, Ross Harper, Tobias U Hauser, Max Rollwage

**Affiliations:** 1 Limbic Ltd London United Kingdom; 2 Department of Psychology King's College London London United Kingdom; 3 Everyturn Mental Health Gosforth United Kingdom; 4 Max Planck UCL Centre for Computational Psychiatry and Ageing Research University College London London United Kingdom; 5 Department of Psychiatry and Psychotherapy Medical School and University Hospital Eberhard Karls University of Tubingen Tübingen Germany; 6 German Center for Mental Health Tübingen Germany

**Keywords:** artificial intelligence, National Health Service, NHS Talking Therapies, mental health, therapy support tool, cognitive behavioral therapy, CBT, chatbot, conversational agent, clinical, patient engagement, therapist, treatment, medication, depression, anxiety disorder, exercise, observational study, control group, patient adherence

## Abstract

**Background:**

Cognitive behavioral therapy (CBT) is a highly effective treatment for depression and anxiety disorders. Nonetheless, a substantial proportion of patients do not respond to treatment. The lack of engagement with therapeutic materials and exercises between sessions, a necessary component of CBT, is a key determinant of unsuccessful treatment.

**Objective:**

The objective of this study was to test whether the deployment of a generative artificial intelligence (AI)–enabled therapy support tool, which helps patients to engage with therapeutic materials and exercises in between sessions, leads to improved treatment success and patient treatment adherence compared with the standard delivery of CBT exercises through static workbooks.

**Methods:**

We conducted a real-world observational study of 244 patients receiving group-based CBT in 5 of the United Kingdom’s National Health Service Talking Therapies services, comparing 150 (61.5%) patients who used the AI-enabled therapy support tool to 94 (38.5%) patients who used the standard delivery of CBT exercises. The groups were equivalent with respect to the content of the CBT materials and the human-led therapy sessions; however, the intervention group received support from the AI-enabled therapy support tool in conducting CBT exercises.

**Results:**

Patients using the AI-enabled therapy support tool exhibited greater attendance at therapy sessions and fewer dropouts from treatment. Furthermore, these patients demonstrated higher reliable improvement, recovery, and reliable recovery rates when compared to the control group, which was related to the degree of use of the AI-enabled therapy support tool. Moreover, we found that engagement with AI-supported CBT interventions, relative to psychoeducational materials, predicted better treatment adherence and treatment success, highlighting the role of personalization in the intervention’s effectiveness. To investigate the mechanisms of these effects further, we conducted a separate qualitative experiment in a nonclinical sample of users (n=113). Results indicated that users perceived the AI-enabled therapy support tool as most useful for discussing their problems to gain awareness and clarity of their situation as well as learning how to apply coping skills and CBT techniques in their daily lives.

**Conclusions:**

Our results show that an AI-enabled, personalized therapy support tool in combination with human-led group therapy is a promising avenue to improve the efficacy of and adherence to mental health care.

## Introduction

### Background

Mental health conditions are a leading cause of health-related burden across the world, with anxiety and depression being ranked among the top 25 causes of burden in 2019 [1]. Both disorders are very common, affecting >29% of the global population in their lifetime [2]. The recent COVID-19 pandemic has accelerated this even further, raising the global prevalence by 27.6% between 2020 and 2021 for major depressive disorder and 25.6% for anxiety disorders [3], highlighting the need for effective and accessible mental health support.

Cognitive behavioral therapy (CBT) has been shown to be a highly effective treatment for depression and anxiety disorders [4-6] and is the first line of treatment for these conditions in many therapy guidelines worldwide [7,8]. Nonetheless, CBT does not benefit everyone. In fact, a recent meta-analysis of 409 trials reported that 58% of patients do not respond to treatment [5]. Several reasons for unsuccessful therapy have been identified. Among them, the most prominent is a failure to engage with therapeutic exercises and materials (ie, homework assignments) between therapy sessions [9].

Between-session support, such as completion of therapeutic materials and exercises, is a necessary component of CBT [10], allowing patients to maintain the progress made in the therapy and transfer it to real life to achieve short-term and long-term improvements [11]. Broadly, between-session work can be divided into psychoeducational (eg, reading materials about symptomatology), self-assessment (eg, monitoring feelings, thoughts, or behaviors), and modality-specific tasks (eg, exposure to images for specific phobias). Meta-analyses have consistently demonstrated that both the quantity and the quality of such task completion significantly predict treatment outcomes [12,13].

Thus, enhancing high-quality engagement with CBT materials and exercises between therapy sessions is a key objective for improving CBT treatment outcomes [14]. This is particularly critical in group therapy settings, where dropout rates are higher and recovery rates are lower than in individual therapy settings [15-17]. Traditionally, therapeutic materials and exercises in CBT have been distributed as worksheets in pen and paper format, downloadable PDF format, or delivered as digital applications, requiring the patient to engage with these static materials without any intelligent supervision. However, this method has shown limitations, often resulting in delayed completion or incomplete materials, highlighting the need for innovative solutions to enhance patient compliance and treatment effectiveness. One promising avenue for enhancing between-session support has been the deployment of digital tools [18]. Most notably, recent developments in generative artificial intelligence (AI) and large language models (LLMs), such as ChatGPT, have showcased that AI can enable a truly tailored user experience with interactions nearly indistinguishable from humans [19] and lead to increased engagement and treatment adherence [20,21]. Thus, the use of this novel technology holds great potential to support clinical practice, especially for supporting patients between therapy sessions to meaningfully engage with their course of treatment.

### Objectives

We developed a generative AI–enabled therapy support tool, Limbic Care, to support patients between sessions during clinician-led group-based CBT. The AI-enabled therapy support tool includes chat-based therapeutic materials to allow for more natural engagement with these materials than traditional, static CBT materials. Recognizing the important role of personalization in overcoming the barrier to the uptake of digital solutions [22], our solution uses generative AI to deliver a user-centric experience. Personalization addresses the diverse needs and preferences of individuals undergoing CBT rather than using the one-size-fits-all approach, which often falls short in addressing unique needs. It may also help cultivate a relationship between users and the AI-enabled therapy support tool. This relationship, also known as therapeutic alliance, has been shown to increase engagement and predict positive clinical change [23]. Thus, we hypothesized that the use of the AI-enabled therapy support tool increases engagement with therapy and thus leads to better outcomes for patients. This support was tailored and personalized, however, treatment was still provided by the clinician in line with medical device regulations [24]. In this observational study, we evaluated a novel solution for patient engagement and clinical outcomes. Assessing 244 patients undergoing CBT treatment in 5 of the United Kingdom’s National Health Service (NHS) Talking Therapies services, we found that patients who chose to use the AI-enabled therapy support tool during their treatment achieved better treatment success. Moreover, we showed that engagement with the AI-enabled therapy support tool was linked to increased reliable improvement, recovery, and reliable recovery, as well as more attended treatment sessions and reduced dropout from treatment. Therefore, our results suggest that the AI-enabled therapy support tool may improve individual patients’ outcomes and support clinical services by increasing treatment adherence and treatment effectiveness.

## Methods

### Setting

We evaluated the efficacy of an AI-enabled therapy support tool (Limbic Care; Limbic Ltd [25]) in a real-world clinical context in 5 NHS Talking Therapies for anxiety and depression services provided by Everyturn Mental Health in the United Kingdom. Specifically, the tool was implemented in the context of completing therapeutic materials and exercises between the sessions of a synchronous web-based group-based CBT program according to the NHS Talking Therapies for anxiety and depression manual [26].

### AI-Enabled Therapy Support Tool

The AI-enabled therapy support tool is a mobile app using AI to offer support between therapy sessions to patients in psychological talking therapy who are aged ≥18 years, under the supervision of a trained clinician.

**Figure 1:**
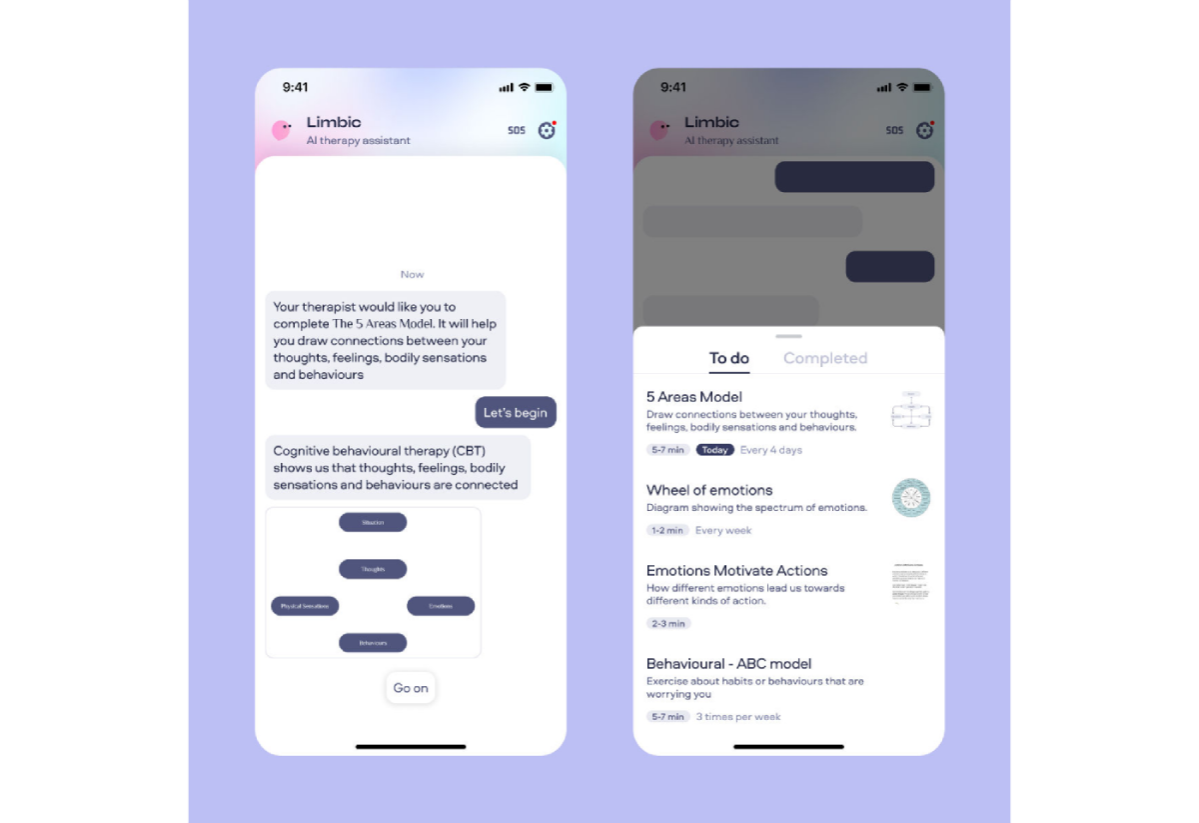
Example user interface of the artificial intelligence–enabled therapy support tool, displaying the conversational interface (left screen) and the assignments provided by the treating clinician (right screen).

The app features a conversational chatbot (Figure 1) to assist with the completion of therapist-assigned therapeutic materials and exercises, which are a component of CBT. The chatbot was powered by an LLM (GPT-4 [OpenAI] at the time of the study) to deliver clinically validated materials, that is, therapeutic materials and exercises developed by clinical experts and assigned by the treating clinician. Specifically, the clinical materials in the AI-enabled therapy support tool were interventions (interactive exercises helping the patient work through a presenting problem, eg, reframing negative thoughts) and psychoeducational materials (educational materials, eg, about CBT). Importantly, there were multiple safety layers and guardrails implemented to ensure that the LLM strictly adhered to the task at hand [27].

The therapeutic materials primarily consist of standard materials for CBT as they are also commonly used in NHS Talking Therapies. These are delivered in a conversational way to the patient using several custom-tailored LLM modules and supervisory AI tools.

All conversations were constantly monitored using several machine learning safety modules to ensure appropriateness, prevent harmful responses, monitor risks, and ensure regulatory compliance [27]. The conversations as well as these machine learning models were monitored and continuously improved by the company’s research team.

In contrast to traditional homework worksheets, the patient can interact conversationally to navigate through the materials and receive notifications regarding assignments. The app encourages and empowers patients to engage in the therapeutic process outside group therapy sessions and continue the progress made in therapy in their real lives as well as provides empathetic support for the user.

### Study Design

This was a multisite, real-world, observational study to investigate the impact of the AI-enabled therapy support tool on clinical outcomes. The AI-enabled therapy support tool was offered to all patients eligible for 11 group-based CBT groups starting between October 2023 and January 2024. All (N=244, 100%) patients had access to the same group sessions irrespective of their decision to use the AI-enabled therapy support tool between the sessions. The clinicians facilitating the group sessions were trained to offer the use of the AI-enabled therapy support tool in the first session of CBT. All (N=244, 100%) patients were encouraged to complete therapeutic materials and exercises between sessions regardless of which delivery method they chose.

We compared the clinical outcomes of individuals who signed up to use the AI-enabled therapy support tool (the intervention group) with those of individuals who did not (the control group). It is important to note that individuals who chose not to use the AI-enabled therapy support tool had access to CBT worksheets, which represent the standard way of completing homework between therapy sessions and which were also assigned by the treating clinician. Therefore, aside from the introduction of the AI-enabled therapy support tool, no other changes were made to the content or the delivery method of the sessions, ensuring that any observed effects can be attributed to patients choosing to use the tool. In addition, we investigated whether engagement with the AI-enabled therapy support tool, specifically the completion of therapeutic materials and exercises on the app, was linked to the outcome measures.

### Ethical Considerations

Clinical audit studies within the NHS Talking Therapies do not require additional patient consent or ethics approval, as determined by the NHS and in line with National Institute for Health and Care Excellence guidelines [28]. This exemption from additional ethical review was further confirmed by the UK Health Research Authority. In this case, a clinical audit was used to determine whether the introduction of a new technology would improve the standard of care. Patients voluntarily opted in to use the AI-enabled therapy support tool. Those who were offered access to the AI-enabled therapy support tool agreed to the privacy and data policy agreement in the app and consented to the use of their anonymized data for audits and research studies. Because this was a real-world observational study in group therapy settings, patients were not provided any financial compensation. Regarding the identification of individuals, treatment and engagement outcome data in this study were anonymous; therefore, it was not possible to identify individual patients.

The qualitative user experience study in a nonclinical sample was separate from the clinical audit and was approved by the University College London Research Ethics Committee (6218/003). Participants in this study were paid £4.20 (US $5.17), and this was not contingent on their use of the AI-enabled therapy support tool. Participation was voluntary, and all users provided informed consent. Free-text answers were anonymized for coding and analysis.

### Outcome Measures

#### Overview

Clinical and app use outcomes were collected as part of the study. Clinical outcomes are routinely assessed throughout treatment provision by NHS Talking Therapies. The measures of treatment engagement include attended sessions, proportion of dropouts, and did not attend (DNA) treatment sessions and the measures of treatment success include reliable improvement, recovery, and reliable recovery [29]. Use outcomes were collected through the mobile app as patients used the AI-enabled therapy support tool and were used as measures of engagement with the intervention. The information collected included the number of sessions in the app and the number of therapeutic materials and exercises completed.

#### Treatment Engagement

##### Number of Therapy Sessions

The number of therapy sessions was used to quantify the total interactions that the patient had with the therapist during their treatment journey. The interactions included assessment and therapy sessions. In NHS Talking Therapies, patients usually have 1 assessment session before the start of treatment and 6 synchronous group therapy sessions. Due to how data are reported by the services, it is not possible to differentiate between assessment and treatment sessions.

##### Proportion of DNA Sessions

DNA sessions refer to sessions that are canceled last minute or not attended by the patient. This was measured as the proportion of DNA sessions out of the total number of sessions that the patient could attend.

##### Dropouts

Dropouts are measured as the percentage of patients who dropped out during the course of treatment.

#### Treatment Success

##### Reliable Improvement

Reliable improvement refers to a clinically significant improvement in symptoms following a course of treatment and is calculated as the score difference between the first and the last validated clinical questionnaire completed. The types of questionnaires patients complete are tailored to their specific condition. For example, the Patient Health Questionnaire-9 (PHQ-9) [30] is used to measure depression symptom severity, and the Generalized Anxiety Disorder-7 (GAD-7) [31] is used to measure anxiety symptom severity. A clinically significant improvement in symptoms is considered a change score ≥6 for PHQ-9 or ≥4 for GAD-7 [26].

##### Recovery

Recovery is achieved in instances where a patient is defined as a clinical case at the start of treatment and not as a clinical case at the end of the treatment. Clinical caseness refers to a patient with severe enough clinical symptoms to be regarded as a clinical case at the start of treatment. Caseness is measured by the clinical questionnaires and is met when patients score ≥10 on PHQ-9 or ≥8 on GAD-7 [26].

##### Reliable Recovery

Reliable recovery is calculated based on reliable improvement and recovery and, as such, is the most stringent outcome. It is achieved if a patient meets the criteria for reliable improvement, referring to clinically significant improvements in their symptoms, and if the patient moves to recovery. Moving to recovery refers to a patient meeting the criteria for clinical caseness at the start of treatment to not being a clinical case at the end of it [26].

#### Use Outcomes

##### Number of Sessions in the App

A session in the app starts when the AI-enabled therapy support tool app is opened and ends when no activity has been detected for ≥30 minutes. This is measured as the number of sessions in the app and is only available for patients using the AI-enabled therapy support tool between sessions.

##### Number of Completed Therapeutic Materials and Exercises

Completed therapeutic materials and exercises refer to the tasks available in the AI-enabled therapy support tool that were initiated and finalized by the patients. This is measured as the number of completed homework assignments and is only available for patients using the AI-enabled therapy support tool between sessions.

### User Experience

In addition to the impact of the AI-enabled therapy support tool on clinical outcomes, we were further interested in the perceived benefits of the tool and potential areas for improvement. Because it can be challenging to contact patients undergoing treatment and minimize the burden on them, we conducted a separate user study to assess user experience. Therefore, we recruited 113 individuals experiencing depression and anxiety symptoms (PHQ-9 score of ≥5 or GAD-7 score of ≥5) from the web-based platform Prolific [32] and instructed them to install the Limbic Care app. Participation was voluntary, and all (113/113, 100%) participants provided informed consent. Participants were not instructed on how to engage with the AI-enabled therapy support tool but rather were told they could use it as much or as little as they would like for the 4-week period of the study. After 4 weeks, participants were invited to answer some questions about their experience using the app, and only users who had used the app were included in the analysis. To probe the perceived benefits of the AI-enabled therapy support tool, participants were asked if they felt their mental health had benefited from using the app (“What benefits to your mental health (if any) have you gained from using the app?”). To understand potential areas for improvement, participants were asked if they had any feedback on how the app could be improved (“Do you have any feedback or suggestions on how we could improve the Limbic app?”). Participants were reimbursed £4.20 (US $5.24) for completing the study, and this was independent of their use of the AI-enabled therapy support tool.

### Qualitative Analysis

Data were collected asynchronously, using free-text answers from a nonclinical, convenience sample on Prolific (n=113). Participants did not have a mental health diagnosis, though they experienced elevated symptoms of anxiety and depression (as indicated by scores of ≥5 for the PHQ-9 and GAD-7). Data were analyzed using reflexive thematic analysis, taking an inductive approach to coding [33], and a realist epistemological position, assuming that meaning and experience are reflected in language [34]. Two researchers (MR and LMD) independently coded a subset of responses and iteratively cross-checked interpretations and codes. Codes were refined using a comparative method, with new interpretations evaluated against existing ones. Inductive analysis of similar codes produced content themes highlighting potential benefits and areas for improvement in the AI-enabled therapy support tool.

Although the semiautomated nature of data collection precluded purposive sampling for saturation, no new codes emerged in later responses that altered the meaning of earlier findings, indicating data saturation was reached [35]. The themes and supporting participant quotes are presented in the Results section.

### Coder Qualifications

The coding team consisted of MR, a male researcher, and LMD, a female researcher. Both researchers, while predominantly quantitative, were trained in thematic analysis and had previous experience in qualitative research [36].

### Trustworthiness

Credibility was ensured through reflexivity and investigator triangulation [37,38]. Data were collected anonymously and asynchronously, minimizing the influence of researcher characteristics on participant responses. Open-ended prompts encouraged both positive and negative reflections to capture a balanced perspective. While the nature of data collection did not allow methodological triangulation, investigator triangulation was achieved through the involvement of multiple researchers (MR, LMD, JH, and JM). To ensure transferability, we described the study’s context and the participants (thick description) to allow the readers to evaluate whether the findings are transferable to other care contexts [37,38]. Dependability and confirmability were ensured by thoroughly documenting the research steps from the start of the study to the interpretation and reporting of the findings [37,38].

### Statistical Methods

We compared pretreatment data for patients in both groups to ensure that they did not differ based on demographic characteristics (age, gender, ethnicity, and sexual orientation), wait time to treatment, and depression and anxiety baseline symptom scores. In addition, recognizing that different referral methods can influence clinical outcomes, as illustrated by the AI-enabled self-referral tool increasing treatment success [21,39], we also compared the referral methods between groups. A 2-tailed chi-square test was applied for categorical outcomes, and a 2-tailed independent student *t* test was used for continuous outcomes.

Next, clinical outcomes were compared between the intervention and the control group. Linear regression models were run for continuous outcomes (number of attended appointments and proportion of DNA sessions), and logistic regressions were run for categorical outcomes (dropouts, reliable improvement, recovery, and reliable recovery). In both cases, the outcomes of interest were entered as independent variables and the intervention group (intervention group=1; control group=0) as a dependent variable. In addition, we controlled for baseline anxiety and depression symptom scores and the proportion of women and heterosexual participants. For linear regression, we reported regression coefficients, along with the 95% CIs, and for logistic regressions, we reported odds ratios (ORs) along with 95% CIs.

Use outcomes were investigated within the group with access to the AI-enabled therapy support tool. Specifically, we were interested in investigating if there would be any association between patients’ level of engagement with the app and their clinical outcomes, in line with previous work suggesting that the completion of therapeutic materials and exercises and engagement between sessions may lead to an improvement in treatment outcomes [14]. Pearson correlation was used for continuous variables, and point-biserial correlation was used for binary variables.

## Results

### Participants

Patients (N=244) attending regular group therapy in Talking Therapies in the United Kingdom were offered to use our AI-enabled therapy support tool, of which 150 (61.5%) downloaded and used the AI-enabled therapy support tool (intervention group). The remaining 94 (38.5%) patients used static therapeutic materials, which were either in pen and paper format or downloadable PDF format (control group). We followed both groups from enrolling in the study until the end of their therapy (typically 8-10 weeks long). To ensure that the patients used the AI-enabled therapy support tool as intended, we calculated the percentage of individuals who engaged with the therapeutic materials at a certain number of weeks from the start of their therapy (known as app retention). We found that 79.3% (119/150) of the individuals were engaged at week 1, 64% (96/150) were engaged at week 2, 51.3% (77/150) were engaged at week 3, 42% (63/150) were engaged at week 4, 35.3% (53/150) were engaged at week 5, and 19.3% (29/150) were engaged at week 6.

To check for baseline differences, we compared pretreatment clinical and demographic characteristics. The mean ages of the participants in the intervention group (mean 40.3, SD 13.2 years) and the control group (mean 40.5, SD 15.5 years) were not significantly different (t_183_=0.11; *P*=.91) nor was the ethnicity of the participants (intervention: 132/150, 88.7% White participants; control: 78/94, 83% White participants; *χ^2^*_1_=0.4; *P*=.53). However, the groups differed significantly in the proportion of heterosexual individuals (intervention: 129/150, 86% heterosexual individuals; control: 69/94, 73% heterosexual individuals; *χ^2^*_1_=5.4; *P*=.02) and women in the group (intervention: 111/150, 74% women; control: 58/94, 62% women; *χ^2^*_1_=4.2; *P*=.04). Therefore, we controlled for the proportion of heterosexual individuals and women in all subsequent analyses. The 2 groups did not differ in anxiety (b=–0.11; *P*=.81) symptom scores nor in depression scores at baseline (b=−0.56; *P*=.36; Table 1). Nonetheless, we decided to control for baseline depression (PHQ-9) and anxiety (GAD-7) scores in all further analyses, as they are known as prognostic factors for changes in symptom severity [40,41]. Finally, the groups did not differ in the time they waited to start treatment (t_122_=–0.32; *P*=.75) nor the proportion of individuals who referred using the AI-enabled self-referral tool (*χ^2^*_1_=1.2; *P*=.28).

**Table 1:** Baseline characteristics of the therapy support and control groups.

	Intervention group (n=150)	Control group (n=94)	*P* value
Age (y), mean (SD)	40.3 (13.24)	40.5 (15.46)	.91
Gender (women), n (%)	111 (74)	58 (61.7)	.04
Sexuality (heterosexual), n (%)	129 (86)	69 (73.4)	.02
Ethnicity (White), n (%)	132 (88.7)	78 (83)	.53
PHQ-9^a^ baseline, mean (SD)	12.6 (4.62)	12.0 (4.55)	.36
GAD-7^b^ baseline, mean (SD)	12.0 (3.30)	12.1 (3.74)	.81
Wait time to treatment (d), mean (SD)	35.4 (19.6)	34.4 (20.4)	.75
Referral method (AI^c^-enabled self-referrals; %)	73.3	66	.28

^a^PHQ-9: Patient Health Questionnaire-9.

^b^GAD-7: Generalized Anxiety Disorder-7.

^c^AI: artificial intelligence.

### Outcome Data

#### AI-Enabled Therapy Support Improves Treatment Engagement

To assess the impact of the AI-enabled therapy support tool on treatment adherence, we compared whether the intervention group attended more therapy sessions, missed fewer sessions, and showed a lower therapy dropout rate than the control group.

##### Increase in the Number of Therapy Sessions

We evaluated whether the AI-enabled therapy support tool facilitated an overall increase in attended therapy sessions. We found that the intervention group attended significantly more sessions, with an average of 2 more sessions attended compared to the control group (b=1.65; 95% CI 1.051-2.200; *P*<.001), illustrating the AI-enabled therapy support tool’s potential to improve engagement in therapeutic processes.

##### Reduction in the Sessions That Were Not Attended

Further analysis focused on the proportion of sessions not attended by the patients (referred to as DNA sessions) to gain a deeper understanding of treatment engagement. We found that the intervention group showed a significant reduction in the rate of DNA sessions (b=–14.97; 95% CI –21.464 to –8.400; *P*<.001), equating to a 15 percentage point reduction in DNA sessions (Table 2). This indicates that the intervention group was more consistent in attending sessions; therefore, it was more engaged in treatment.

**Table 2:** The percentage point difference between the intervention and control groups for treatment engagement and treatment success measures.

Outcome	Percentage point difference (%)	Group effect (b)	*P* value
DNA^a^ session proportion	–15	–14.97	<.001
Dropout rate	–23	–1.14	<.001
Reliable improvement	21	80	.005
Recovery	25	1.03	.001
Reliable recovery	21	86	.004

^a^DNA: did not attend.

##### Reduction in Dropout From Therapy

More critical than DNA sessions were participants who did not finish their therapy and dropped out completely. Therefore, we investigated whether the AI-enabled therapy support tool was also linked to reduced dropout rates. We found that the intervention group experienced a significant 23 percentage point reduction in dropout rates (OR 0.32, 95% CI 0.171-0.595; *P*<.001; Table 2) compared to individuals in the control group. This suggests that the AI-enabled therapy support tool was associated with an increased likelihood of finishing a course of treatment.

Tables S1 and S2 in Multimedia Appendix 1 further evaluate the impact of the AI-enabled therapy support tool on treatment engagement separately for patients with a primary diagnosis of depression or an anxiety disorder at baseline. Briefly, we found that the tool is equally effective for both conditions.

#### AI-Enabled Therapy Support Improves Treatment Success

**Figure 2:**
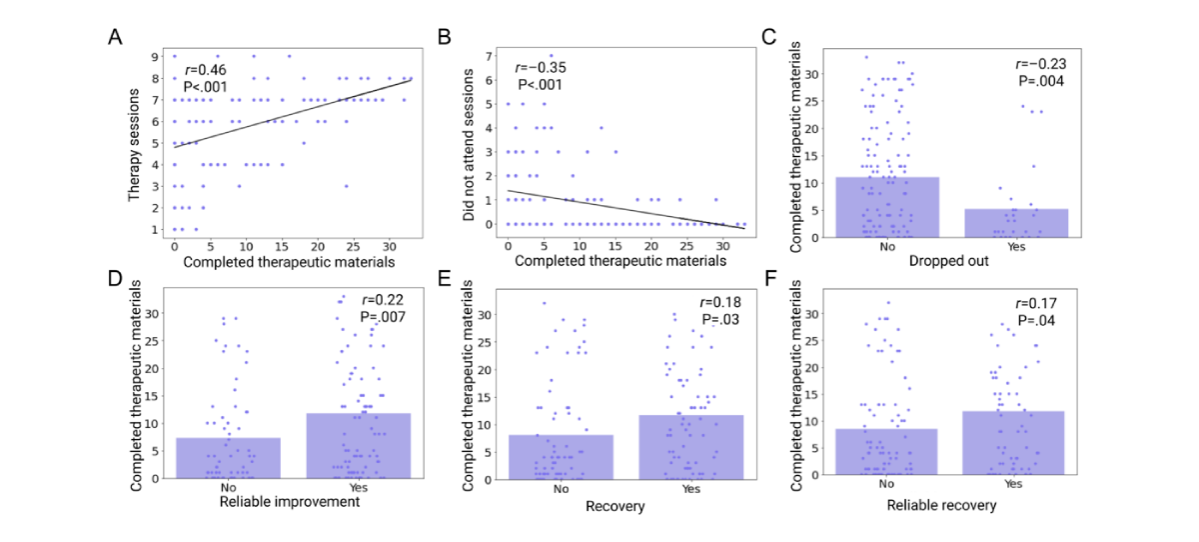
Correlations between completed therapeutic materials, exercise assignments, and treatment engagement (A-C) and treatment success measures (D-F).

Next, to assess the impact of the AI-enabled therapy support tool, we investigated the 3 key metrics for treatment success that are routinely assessed in NHS Talking Therapies [26] (Figure 2).

##### Increased Reliable Improvement

First, we compared the reliable improvement rate between the groups. Reliable improvement is defined as a significant change in patients’ symptom scores from the beginning to the end of treatment (reduction of ≥6 points in the PHQ-9 or ≥4 points in the GAD-7 total scores). This measure is considered to reflect meaningful clinical progress. We found that the intervention group experienced a 21 percentage point higher rate of reliable improvement compared to the control group (Table 2). This difference was statistically significant (OR 2.21, 95% CI 1.279-3.834; *P*=.005), indicating that using the AI-enabled therapy support tool was linked to a higher likelihood that the individual saw a clinical improvement in their symptoms.

##### Improved Recovery

Next, we were interested in recovery, going beyond mere symptom improvement. Therefore, we looked at how many patients moved from caseness (meaning that the patient has severe enough symptoms to be considered a clinical case for depression or anxiety disorders) to below caseness during their treatment, indicating recovery [26]. We found that the intervention group experienced a 25 percentage point higher recovery rate compared to the control group (Table 2). This difference was highly significant between groups (OR 2.81, 95% CI 1.561-5.069; *P*=.001), showing that patients in the intervention group were more likely to recover during the administered therapy.

##### Better Reliable Recovery

Finally, we investigated the most rigorous criteria for treatment success, namely reliable recovery [26]. Reliable recovery is achieved when a patient shows both of the aforementioned criteria, that is, reliable improvement as well as recovery. In other words, the patient has moved from being considered a clinical case to being a nonclinical case and there has also been a significant improvement in their condition.

We found that the intervention group had 21 percentage points higher reliable recovery rates compared to the control group (Table 2). This difference was significant between the 2 groups (OR 2.37, 95% CI 1.311-4.290; *P*=.004), showing that patients in the intervention group were more likely to achieve reliable recovery. In summary, these results show that the people who used the AI-enabled therapy support tool achieved substantially and consistently better treatment outcomes than those who did their homework using traditional means.

Tables S1 and S2 in Multimedia Appendix 1 further evaluate the impact of the AI-enabled therapy support tool on treatment success separately for patients with a primary diagnosis of depression or an anxiety disorder at baseline. Briefly, we found that the tool is equally effective for both conditions.

#### Use of AI-Enabled Therapy Support Tool Linked to Better Treatment Adherence and Success

A crucial aspect of the usefulness of AI-enabled therapy support tools is whether these are being used by the patients. During the course of treatment, patients in the intervention group used the AI-enabled therapy support tool, on average, for 23 sessions. However, there was some variability in use within the intervention group. Therefore, we investigated if higher engagement with the AI-enabled therapy support tool was associated with therapy adherence in a dose-dependent manner.

First, we found a statistically significant link between the number of completed therapeutic exercises in the AI-enabled therapy support tool and the number of therapy sessions (*r*=0.46; *P*<.001; Figure 2A), meaning those who engaged more with the AI-enabled therapy support tool were more likely to attend more therapy sessions. In the same spirit, individuals using the AI-enabled therapy support tool between sessions had a lower proportion of recorded DNA sessions (*r*=–0.35; *P*<.001; Figure 2B), indicating that they were more consistent in attending scheduled sessions. Individuals who completed more tasks on the AI-enabled therapy support tool were also found to be less likely to drop out from the treatment program (*r*_pb_=–0.23; *P*=.004; Figure 2C), suggesting that the tool not only enhanced session attendance but also contributed to sustained treatment participation.

In addition, we found that the higher use of the AI-enabled therapy support tool was associated with better treatment success, such as reliable improvement (*r*_pb_=0.22; *P*=.007; Figure 2D), recovery (*r*_pb_=0.18; *P*=.02; Figure 2E), and reliable recovery (*r*_pb_=0.17; *P*=.04; Figure 2F). Therefore, these results indicated that the tool was effective in promoting both treatment adherence and clinical treatment efficacy.

Beyond the number of completed therapeutic materials, we were also interested in other measures of engagement with the AI-enabled therapy support tool to gain a more in-depth understanding of the link between app activity and treatment outcomes. As additional engagement measures, we investigated the overall app sessions and total time spent using the app.

We found statistically significant associations between the total number of sessions in the app and dropout rate (*r*=–0.25; *P*=.007) and increased reliable improvement (*r*=0.23; *P*=.01), recovery (*r*=0.20; *P*=.02), as well as reliable recovery (*r*=0.17; *P*=.046). The total number of sessions spent in the app was not correlated with attended appointments (*r*=0.13; *P*=.20) nor DNA sessions (*r*=–0.14; *P*=.18).

Finally, we were also interested in assessing potential links between time spent in the app with clinical and engagement outcomes. Similar to the results mentioned earlier, we found statistically significant associations between time spent in the app and dropout rate (*r*=–0.25; *P*=.007), increased reliable improvement (*r*=0.23; *P*=.01), recovery (*r*=0.20; *P*=.02), as well as the more stringent metric of reliable recovery (*r*=0.17; *P*=.046). Time spent in the app was not associated with attended appointments (*r*=0.14; *P*=.18) nor DNA sessions (*r*=–0.12; *P*=.21).

#### Higher Engagement with CBT Exercises Relative to Psychoeducation Predicts Better Treatment Adherence and Success

**Figure 3:**
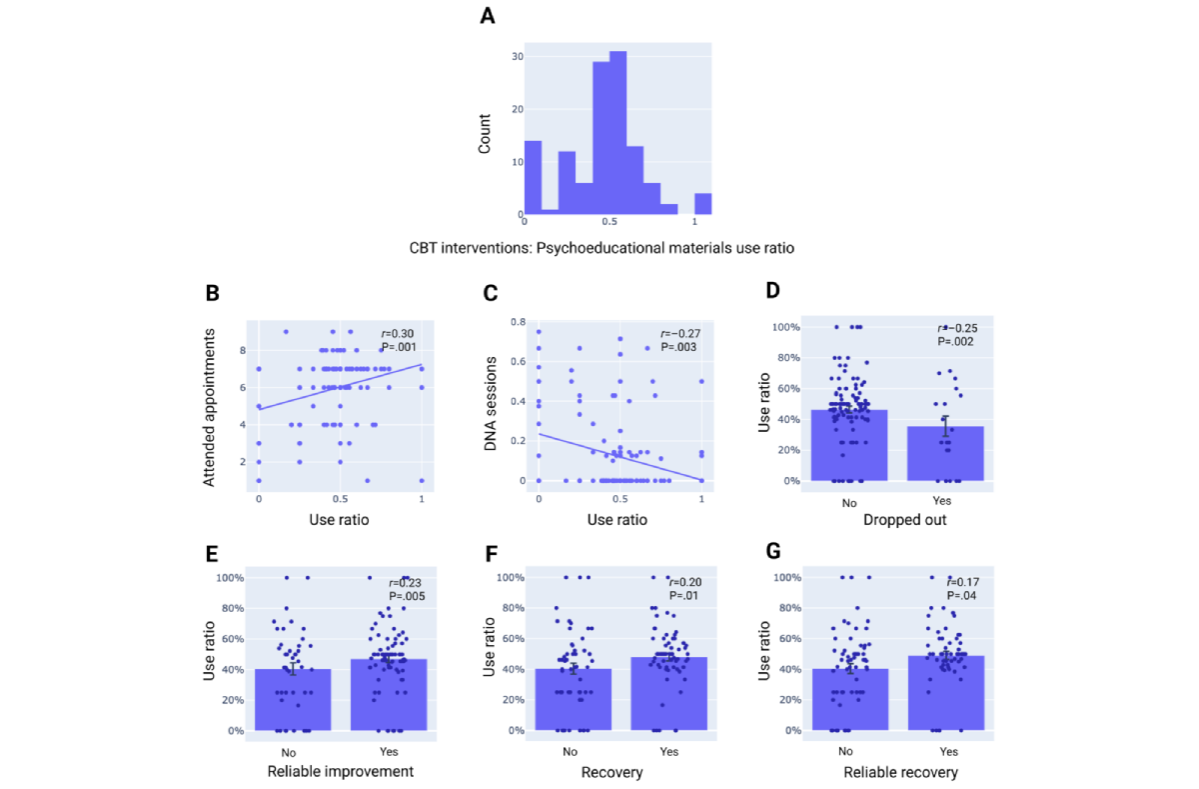
Use ratio of cognitive behavioral therapy interventions relative to psychoeducational materials (A), correlations between use ratio and treatment engagement outcomes (B-D), and correlations between use ratio and treatment success outcomes (E-G). Error bars indicate standard error of the mean. CBT: cognitive behavioral therapy; DNA: did not attend.

Of the 150 patients in the intervention condition, 118 (78.7%) completed at least 1 AI-supported CBT intervention (an interactive CBT exercise helping patients work through a problem) or psychoeducational material (educational material about CBT). Of these, 4 (2.7%) patients only completed interventions, and 14 (9.3%) patients only engaged with psychoeducational materials. The remaining 100 (66.7%) patients engaged with at least 1 CBT intervention and 1 psychoeducational material (number of interventions: mean 6.7, SD 4.78; number of psychoeducational materials: mean 6.5, SD 4.22). In this subgroup of 100 patients, we evaluated whether engagement with a specific type of AI-supported therapeutic material was predictive of treatment outcomes. To this end, we computed a use ratio of the number of CBT exercises to the number of psychoeducational materials completed (Figure 3A). We found that completing more CBT exercises, relative to psychoeducational materials, predicted better treatment adherence, for example, increased the number of attended appointments (*r*=0.30; *P*=.001) and reduced the proportion of DNA sessions (*r*=–0.27; *P*=.003) as well as the dropout rate (*r*=–0.25; *P*=.002; Figures 3B-3D). Furthermore, the increased use of CBT interventions was also associated with an improvement in treatment success, for example, increased reliable improvement (*r*=0.23; *P*=.005), recovery (*r*=0.20; *P*=.01), and reliable recovery (*r*=0.17; *P*=.04; Figures 3E-3G). This suggests that especially the engagement with the personalized CBT exercises delivered through generative AI was associated with treatment adherence and treatment success compared to engagement with psychoeducational CBT materials.

### Other Analyses

#### User Experience

Beyond the investigation of the effects of the AI-enabled therapy support tool on treatment success and treatment adherence outcomes, we were also interested in conducting a qualitative investigation into the perceived benefits of using this AI-enabled therapy support tool as well as potential areas for improvement. Investigating potential benefits in more depth provides the opportunity to understand the mechanism of why the AI-enabled therapy support tool might have advantages over standard care.

Therefore, in a separate user experience study, 113 participants downloaded and used the Limbic Care app for 4 weeks and provided free-text answers on perceived benefits related to using the app as well as on potential areas for improvement. The sample had a mean age of 31.32 (SD 8.72) years and consisted of 53.1% (60/113) women. A total of 47 (41.6%) participants identified as White. Participants in our nonclinical sample experienced some symptoms of depression and anxiety (PHQ-9 score of ≥5 or GAD-7 score of ≥5); their scores were notably lower than the patient sample, as expected (PHQ-9: mean 10.15, SD 6.14; GAD-7: mean 8.74, SD 4.64).

#### Perceived Benefits

Of the 113 users surveyed, 92 (81.4%) mentioned a specific benefit of the AI-enabled therapy support tool to their mental health. Here, the most mentioned benefit (30/113, 26.5%) was improved awareness and clarity, indicating that the conversations with the AI-enabled therapy tool were especially helpful for users to think through their issues and gain better perspectives on their situation. The second most frequently reported benefit (23/113, 20.4%) consisted of coping strategies and CBT techniques, suggesting that AI-enabled support helped patients understand and apply CBT exercises to their day-to-day lives. A total of 14 (12.4%) users reported the positive benefit of mindfulness and relaxation, indicating that immediate support for dealing with stressful situations and helping to reduce emotional responses were important aspects of the tool. In addition, 9 (8%) users mentioned empathetic and nonjudgmental support as the main benefit, suggesting that a place to vent and be heard when no human is available was an important component of the tool. Finally, 15 (13.3%) users reported a broad positive impact on their mental health without focusing on any of these more specific categories.

### Opportunities for Refinement

The themes and supporting participant quotes are presented in Table 3.

Of the 113 users, only 38 (33.6%) had suggestions for areas of potential improvement. The rest of the users had either no suggestions for improvement (n=63, 55.8%) or mentioned positive feedback even when queried about areas of improvement (n=12, 10.6%). Of the users who had suggestions for improvements, the most common comment (17/113, 15%) focused on the fact that while the chatbot was already very personalized, it was still possible to detect that it was not a human. In addition, 11 (9.7%) users had suggestions for specific additional features that they would either like to see or did not enjoy in the app. There were 4 (3.5%) users asking for more tailored notifications, and 3 (2.7%) users found that the additional guard rails to ensure patient safety restricted their user experience.

This indicated that, overall, users suggested few areas for improvement, with many of these dimensions being highly idiosyncratic to individual users.

**Table 3:** Overview of themes relating to the perceived benefits of using the artificial intelligence (AI)–enabled therapy support tool.

Theme	Definition	Example quotes
Emotional support and empathetic listening	The AI-enabled therapy support tool provided nonjudgmental and empathetic support, which enabled participants to open up about their problems even in the absence of another human being available to discuss their problems.	“Limbic has helped me take the time to be grateful for things in my life, and to take the time to appreciate things about myself—even if they’re small things.... I have done a couple of the exercises in the app, and they are helpful as well. I feel like I have learned new ways to cope with my mental health. It’s also just really nice just openly talking to an AI who doesn’t judge your feelings or anything.” “I feel more empowered to talk to my doctor and partner about my low mood and lack of energy. I like that I can talk to the chatbot when I feel like I can’t control my emotions, and it helped having someone to be completely honest with.”
Awareness, clarity, and new perspectives	The AI-enabled therapy support tool supported participants to think through their problems, identify their patterns, and come up with new perspectives on certain issues, resulting in improved clarity about feelings or problems.	“I feel like I have gained some self-awareness and also that I now have tools and strategies to deal more effectively with my negative emotional states.” “The act of putting my thoughts and feelings into words... helps bring clarity to them for me.” “Thinking of how I should ground myself when I feel really overwhelmed. Trying to put things in a different perspective outside of my own head.”
Coping strategies and CBT^a^ techniques	The AI-enabled therapy support tool supported participants by providing information about CBT techniques and exercises, which were helpful to apply to the users’ problems, helping them to better cope with their mental health.	“I’ve been able to see the positive changes that come from opening up to someone and seeking advice on the issues that I’m dealing with. Each time that I have opened up in the Limbic app, it has provided me with a safe space, reassurance, it acknowledges me and validates me, and always provides me with options for strategies that might be helpful to me. I am very grateful to have an app like this on my phone!” “I learned some new methods on how to deal with some of the problems I have.”
Mindfulness and relaxation	The AI-enabled therapy support tool supported participants to be more mindful of their emotions and supported them in dealing with stressful situations, resulting in feeling more relaxed and balanced.	“More self-awareness and mindfulness. Reminders to check in on myself and be kind to myself.”
Gratitude	The AI-enabled therapy support tool supported participants to be more mindful and grateful for the positive experiences in their lives.	“Setting and keeping track of my goals and being thankful for small things, thanks to the journaling activity.” “It has helped me acknowledge that there are good things in my life to be grateful for. It helped me realise that I do not need to only focus on what is going wrong.”
General positive impact	The AI-enabled therapy support tool had generally broad positive impact on participants’ mental health	“It has improved my mental health.” “I think it helped to reduce my stress and my anxiety and enhanced my overall well-being.”

^a^CBT: cognitive behavioral therapy.

## Discussion

### Principal Findings

We investigated the effects of a generative AI–enabled therapy support tool on treatment success and treatment adherence for patients undergoing group therapy in 5 of the UK NHS Talking Therapies provided by Everyturn Mental Health. A key part of therapy is the engagement with therapeutic materials that are assigned to the patient by the therapist. This engagement is crucial for applying tools and techniques learned in the therapy to real-life contexts [10]. In our study, the intervention group received in-between session support through an AI-enabled therapy support tool, while the control group completed the assignments using standard, static CBT worksheets.

Patients who had access to the AI-enabled therapy support tool showed significant improvement in clinical outcomes. Specifically, they achieved higher rates of reliable improvement, recovery, and reliable recovery compared to the control group. Similarly, patients using the AI-enabled therapy support tool attended more therapy sessions, were less likely to not show up to scheduled therapy sessions (that is, fewer DNAs), and were less likely to drop out from therapy. Importantly, the tool’s use level was significantly associated with treatment success. Particularly, higher engagement with the AI-enabled therapy support tool was associated with increased reliable improvement and better recovery and reliable recovery. Furthermore, the use of the AI-enabled therapy support tool was significantly associated with treatment adherence, that is, app use was correlated with a higher number of attended therapy sessions and with a lower proportion of missed appointments and treatment dropouts. Therefore, our findings suggest that integrating the AI-enabled therapy support tool into standard therapy may lead to positive outcomes at the individual level by improving clinical outcomes. Moreover, the tool may save precious clinical time by reducing missed appointments and through this may reduce costs for the services.

### Interpretation

Our findings indicate that using the AI-enabled therapy support tool to support patients between sessions can improve treatment outcomes. In line with previous research indicating that more meaningful engagement leads to increased intervention effectiveness [42] and hence better treatment success [43], we found that the use of the AI-enabled therapy support tool was associated with increased reliable improvement, recovery, and reliable recovery–the 3 core metrics for treatment success that are routinely assessed in NHS Talking Therapies [26]. One key aspect of our AI-enabled therapy support tool is personalization, a crucial component of CBT [44], which means that patients receive tailored information and interactions to support them in understanding how the CBT exercises relate to their situations and daily problems. Cultivating a therapeutic relationship or therapeutic alliance has been shown to increase engagement and predict positive clinical change [23]. In the case of our AI-enabled therapy support tool, we encouraged this using an interactive chat with the patients, which provided personalized and individualized assistance. It is noteworthy that these effects seem to be specifically linked to the delivery of CBT exercises through generative AI rather than only by displaying CBT psychoeducation materials in a digitized format per se*.* Moreover, the qualitative analysis of user feedback suggests that these generative conversations were especially useful for helping users explore new perspectives and views on their problems, resulting in increased clarity. These findings suggest a unique role of generative AI in the delivery of CBT exercises.

It is also worth noting that higher engagement with the app was linked to more attended sessions and a lower proportion of reported DNA sessions. This indicates that increased engagement between sessions is not only beneficial to the individual patients but also to the services that experience approximately 12% fewer missed appointments and 42% fewer treatment dropouts [45]. Furthermore, considering the substantial cost per reliable recovery in low-intensity treatment in NHS Talking Therapies, estimated at £1087 (US $1355.91) [46], the increase of 21 percentage points observed in the AI-enabled therapy support tool group translates to substantial cost savings. Assuming that the AI-enabled therapy tool is solely driving this 21% improvement, it would translate to £228 (US $284.40) of generated value per patient based on improved recovery alone, making it highly cost-effective. While future studies should assess the cost-effectiveness of such tools in detail, our data indicate that enhancing treatment effectiveness within NHS Talking Therapies through AI-enabled therapy support could lead to substantial cost savings and improved quality of care.

There has been a large societal debate about the use of AI, and especially LLMs, in clinical settings [47], emphasizing the importance of caution when introducing novel technologies into health care settings. Importantly, the AI-enabled therapy support tool has undergone rigorous testing as part of an International Organization for Standardization 13485–compliant quality management system, ensuring the product has gone through gold-standard risk management processes. Moreover, the AI-enabled therapy support tool was only complementing standard care provided by trained clinicians. Thus, the AI-enabled therapy support tool is only populated with therapeutic materials chosen by the clinician rather than making any clinical decisions itself. While this is a low-risk task, the positive impact on treatment outcomes was nevertheless substantial. These findings exemplify the importance of critically evaluating how AI can be used safely and effectively in clinical settings.

### Generalizability

Importantly, our study uses data acquired from real patients receiving treatment from the UK NHS Talking Therapies, which means that the presented data provide high ecological validity for real-world clinical settings. Another advantage of our study is that the AI-enabled therapy support tool offered personalization through generative AI, and a lack of personalization has been identified as a key barrier to the uptake of digital solutions in previous research [22]. Lastly, the novel AI-enabled therapy support tool targeted support between therapy sessions, which is one of the most prominent reasons for disengagement [9].

### Limitations

First, it is important to consider the observational design of this study. Following this promising observational data, a randomized controlled trial should be conducted. Second, while we were statistically controlling for several potential confounding factors (eg, patients’ symptom severity), we did not have access to and were therefore unable to control for certain aspects, such as socioeconomic status. Nonetheless, given that the participants in both groups were from the same services, and thus similar geographic areas, we are not expecting major differences in their socioeconomic background. Studies with randomized designs may help to control for such confounds and would be a valuable future research direction. Finally, while we incorporated qualitative feedback about the perceived benefits of the AI-enabled therapy support tool, as well as potential areas where the tool could be improved, these data were collected from a nonclinical convenience sample of users. While the users showed elevated levels of depression and anxiety symptoms, it is possible that their views do not align with those of the patient sample. To this end, we would aim to incorporate a mixed methods process evaluation as part of a future randomized controlled trial evaluating the effectiveness of the tool.

### Conclusions

In conclusion, this study showed that integrating an AI-enabled therapy support tool can lead to positive outcomes for patients and clinical services alike, by improving therapeutic outcomes and reducing costs associated with missed or dropout sessions. These are important findings considering that AI-enabled digital mental health tools are an emerging field and that they have the potential to increase access to treatment for patients and lessen the burden on mental health services.

## Appendix

Multimedia Appendix 1 App user interface, correlations between app engagement and treatment outcomes, and use ratio results.

## Abbreviations

AI: artificial intelligence
CBT: cognitive behavioral therapy
DNA: did not attend
GAD-7: Generalized Anxiety Disorder-7
LLM: large language model
NHS: National Health Service
OR: odds ratio
PHQ-9: Patient Health Questionnaire-9

## Data Availability

All analyses were based on sensitive clinical data, which we are not permitted to share with third parties. Summary statistics, as displayed in figures and text, are available upon request from the corresponding author.
