# Supplementary material for: Generative AI–Enabled Therapy Support Tool for Improved Clinical Outcomes and Patient Engagement in Group Therapy: Real-World Observational Study
Source: J Med Internet Res. 2025 Mar 10;27:e60435. doi: 10.2196/60435 (PMC11933774; doi:10.2196/60435)
Supplement: Multimedia Appendix 1 [file jmir_v27i1e60435_app1.docx]

**Supplementary materials**

Generative AI-Enabled Therapy Support Tool Improves Clinical Outcomes and Patient Engagement in NHS Talking Therapies

Johanna Habicht*^1^, Larisa-Maria Dina*^1,2^, Jessica McFadyen^1^, Mona Stylianou^3^, Ross Harper^1^, Tobias U. Hauser^1,4,5,6^, Max Rollwage^1^

To further investigate if the AI-enabled therapy support tool might be more effective in individuals with a primary diagnosis of anxiety or depression at baseline, the following checks were conducted: (1) we reported the distribution of primary diagnoses across the intervention and control conditions at baseline and investigated potential differences using a chi-squared test; (2) we ran logistic (categorical outcome ~ group*diagnosis + sexuality + gender + phq9_start + gad7_start) and linear (continuous outcome ~ group*diagnosis + sexuality + gender + phq9_start + gad7_start) regressions to investigate potential interactions between the treatment group and primary diagnosis at baseline. The full models are reported in Table S2 below.

***Distribution of diagnoses at baseline***

| ***group*** | ***diagnosis*** | ***count*** | ***proportion*** |
| --- | --- | --- | --- |
| *control* | *anxiety* | *48* | *51.06%* |
| *control* | *depression* | *44* | *46.81%* |
| *control* | *other* | *2* | *2.13%* |
| *intervention* | *anxiety* | *80* | *53.33%* |
| *intervention* | *depression* | *64* | *42.67%* |
| *intervention* | *other* | *6* | *4.00%* |

***Table S1.*** *Distribution of diagnoses at baseline across treatment groups (intervention versus control).*

The distribution of anxiety and depression diagnoses at baseline was similar across treatment groups (Table S1), χ² = 0.140, *p* = 0.708. Patients with a primary diagnosis other than anxiety or depression (denoted as ‘other’) were excluded from the analysis.

***Effectiveness of the AI-enabled therapy support tool for each diagnosis***

| **Outcome** | **Group effect (β, p-value)** | **Group x diagnosis effect (β, p-value)** |
| --- | --- | --- |
| Attended appointments | β = 1.399  p = 0.001** | β = 0.055  p = 0.929 |
| DNA proportion | β = -0.139  p = 0.003** | β = 0.035  p = 0.607 |
| Drop-out | β = -1.467  p = 0.002** | β = 0.917  p = 0.155 |
| Reliable improvement | β = 1.087  p = 0.006** | β = -0.697  p = 0.222 |
| Recovery | β = 1.219  p = 0.003** | β = -0.538  p = 0.381 |
| Reliable recovery | β = 1.033  p = 0.011* | β = -0.617  p = 0.317 |

**Table S2**. Logistic and linear regression results investigating potential interactions between treatment groups (intervention versus control) and diagnosis (anxiety disorder and depression).

We find significant main effects of group (intervention versus control) for all outcome measures even after controlling for diagnosis, sexuality, gender, PHQ-9 and GAD-7 scores at baseline. Moreover, no significant interaction between treatment group and diagnosis was observed for any of the outcome measures. This indicates that the AI-enabled therapy support tool is equally effective in both diagnosis groups (anxiety disorders and depression).”
